# Supplementary material for: A Likelihood Approach for Real-Time Calibration of Stochastic Compartmental Epidemic Models
Source: PLoS Comput Biol. 2017 Jan 17;13(1):e1005257. doi: 10.1371/journal.pcbi.1005257 (PMC5240920; doi:10.1371/journal.pcbi.1005257)
Supplement: S1 File — (TAR.GZ) [file pcbi.1005257.s014.tar.gz › HSPH_Online-SI-Revision/IPoi09/n100-severe/n100-severe_BENCH_summary.pdf]

peak -8	R0	$1/\theta_2$	inf	inflog	newly	newlylog	Reff	AttackRate
mean	27.2	71.7	211.3	0.5	325.9	0.7	29.9	33.6
median	25.6	57.2	156.9	0.5	222.6	0.6	29.	34.6
Quantile .1	6.4	31.3	76.5	0.3	71.8	0.4	9.4	8.7
Quantile .9	43.4	135.5	338.6	0.8	815.5	1.	49.2	47.1
21	18	20						
length	50							

peak -4	R0	$1/\theta_2$	inf	inflog	newly	newlylog	Reff	AttackRate
mean	25.4	65.8	161.1	0.4	110.8	0.4	28.6	21.6
median	14.3	37.6	157.2	0.4	113.1	0.4	20.7	18.
Quantile .1	2.4	9.9	79.7	0.3	52.7	0.3	11.4	4.
Quantile .9	69.8	167.5	227.3	0.5	186.6	0.5	66.1	45.4
1	2	3						
length	50							

peak	R0	$1/\theta_2$	inf	inflog	newly	newlylog	Reff	AttackRate
mean	78.5	160.7	227.6	0.5	37.6	0.1	41.3	48.4
median	78.2	153.4	205.	0.5	33.4	0.1	41.4	45.
Quantile .1	62.6	82.7	148.4	0.4	18.5	0.1	35.7	34.6
Quantile .9	96.2	235.5	352.3	0.6	65.	0.2	45.6	65.9
0	0	0						
length	50							

peak +4	R0	$1/\theta_2$	inf	inflog	newly	newlylog	Reff	AttackRate
mean	79.3	161.1	526.1	0.7	157.9	0.3	52.8	48.6
median	78.2	154.8	522.1	0.7	143.	0.3	52.4	45.4
Quantile .1	67.1	101.9	200.7	0.5	60.1	0.2	44.6	35.2
Quantile .9	90.7	240.7	808.8	0.9	266.	0.5	60.5	65.9
0	0	0						
length	50							

peak +8	R0	$1/\theta_2$	inf	inflog	newly	newlylog	Reff	AttackRate
mean	73.9	146.2	1585.3	0.9	635.4	0.6	58.7	47.2
median	74.3	145.3	871.3	0.9	268.3	0.5	59.8	44.7
Quantile .1	63.3	102.5	279.3	0.5	94.2	0.2	51.5	34.8
Quantile .9	81.9	189.2	1901.6	1.2	689.2	0.8	64.3	63.8
0	0	0						
length	50							
